# Animal Models of Human Pathology 2013

**DOI:** 10.1155/2014/861424

**Published:** 2014-02-20

**Authors:** Monica Fedele, Oreste Gualillo, Andrea Vecchione

**Affiliations:** ^1^Institute of Experimental Endocrinology and Oncology (IEOS), CNR, 80131 Naples, Italy; ^2^Santiago University Clinical Hospital IDIS, 15706 Santiago de Compostela, Spain; ^3^Human Cancer Genetics Program, Division of Pathology, Comprehensive Cancer Center, The Ohio State University, Columbus, OH 43216, USA

Despite the success of biomedical engineering that made it possible to reproduce *in vitro* whole organ systems, biological processes are complex and cannot be replaced or properly simulated *in vitro* or *in silico*. Therefore, medicine still needs *in vivo* studies in whole organisms to ensure that the scientific demonstrations are as close as possible to human life.

In this special issue we collected six reviews and nine research articles describing studies using animals as disease models. In particular, you will read about the development and use of animal models for new therapeutic strategies, as well as for the development of the scientific understanding, related to different human pathologies, including cancer, autism, osteoarthritis, cardiovascular diseases, secondary lymphedema, metabolic syndrome, and abnormal scarring.

Several contributions focus on cancer: M. Lamberti et al. discuss about the different mechanisms of cardiotoxicity induced by antiblastic drugs assessed using animal models; S. Bimonte et al. review the role of morphine in primary tumor growth in animal models, focusing upon invasiveness and development of metastasis; the same group also presents a nice contribution showing, in an orthotopic mouse model of pancreatic cancer, that curcumin has a great potential in treatment of human pancreatic cancer, through the modulation of NF-*κ*B pathway; M. Carvalho et al. review the role of T-lymphocytes in breast cancer, both in human and canine models; C. Calixto-Campos et al. describe a novel model of cancer pain for pathophysiological studies and pharmacological screening.

Four research articles are interested in the topic of cardiovascular diseases: N. E. Beltran et al. analyze the gastric tissue damage induced by ischemia, using a bioimpedance confocal endomicroscopy; G. Biondi-Zoccai et al. report the design and ensuing application of a novel porcine experimental model of closed-chest chronic ischemia, suitable for biomedical research, mimicking postmyocardial infarction heart failure; T. Spata et al. tackle the development of a platelet-induced coronary occlusion in a large animal, such as the sheep; Y. Cheng et al. show, in ovariectomized rats, that estradiol attenuates the decreased hippocampal neurogenesis produced by poststroke depression.

A new promising therapy for the autism is reviewed by A. C. Santini et al. who describe the treatment with the NMDAR glycine site against GLYX-13 for the therapy in autistic children, whereas the metabolic syndrome is the topic of V. Venkatesan et al. who evaluate the pancreatic features in a peculiar obese rat model (WNIN/Ob), showing that several cofounding factors in the pancreatic milieu take part to a profound state of inflammation in pancreas, leading to the onset of obesity/insulin resistance, which get worsened with age.

Finally, three papers describe animal models for three different human pathologies: B. F. Seo et al. review the current status of animal models of keloid and hypertrophic scarring; H. S. Park et al. show interesting data sets in creating an improved rodent hindlimb model of secondary lymphedema; M. Moreau et al. optimize the dog model of experimental osteoarthritis and show how the combination of this model with the dog model of naturally-occurring osteoartritis has the potential to be translated to the human condition, offering the opportunity to investigate structural and functional benefits of disease modifying strategies.

We hope that the articles in this special issue will be of interest to the scientific community, providing new elements for the study and treatment of some human diseases.

Lastly, we would like to thank all the authors for their efforts and all the reviewers for their critical notes useful to improve the papers.

*Monica Fedele* *Oreste Gualillo* *Andrea Vecchione*

